# Thickness scaling of atomic-layer-deposited HfO_2_ films and their application to wafer-scale graphene tunnelling transistors

**DOI:** 10.1038/srep20907

**Published:** 2016-02-10

**Authors:** Seong-Jun Jeong, Yeahyun Gu, Jinseong Heo, Jaehyun Yang, Chang-Seok Lee, Min-Hyun Lee, Yunseong Lee, Hyoungsub Kim, Seongjun Park, Sungwoo Hwang

**Affiliations:** 1 Device Laboratory, Device and System Research Center, Samsung Advanced Institute of Technology, Suwon 443-803, Korea; 2 School of Advanced Materials Science and Engineering, Samsung-SKKU Graphene/2D Center, Sungkyunkwan University, Suwon 440-746, Korea

## Abstract

The downscaling of the capacitance equivalent oxide thickness (CET) of a gate dielectric film with a high dielectric constant, such as atomic layer deposited (ALD) HfO_2_, is a fundamental challenge in achieving high-performance graphene-based transistors with a low gate leakage current. Here, we assess the application of various surface modification methods on monolayer graphene sheets grown by chemical vapour deposition to obtain a uniform and pinhole-free ALD HfO_2_ film with a substantially small CET at a wafer scale. The effects of various surface modifications, such as N-methyl-2-pyrrolidone treatment and introduction of sputtered ZnO and e-beam-evaporated Hf seed layers on monolayer graphene, and the subsequent HfO_2_ film formation under identical ALD process parameters were systematically evaluated. The nucleation layer provided by the Hf seed layer (which transforms to the HfO_2_ layer during ALD) resulted in the uniform and conformal deposition of the HfO_2_ film without damaging the graphene, which is suitable for downscaling the CET. After verifying the feasibility of scaling down the HfO_2_ thickness to achieve a CET of ~1.5 nm from an array of top-gated metal-oxide-graphene field-effect transistors, we fabricated graphene heterojunction tunnelling transistors with a record-low subthreshold swing value of <60 mV/dec on an 8″ glass wafer.

Graphene, a two-dimensional (2D) monolayer composed of sp^2^ bonded carbon atoms in a hexagonal arrangement, has been shown to exhibit exceptional electrical, optical, thermal, and mechanical properties, which has generated a significant number of studies exploring its application to various nanoelectronic devices^1^,^2^. Owing to its extremely high charge carrier mobility originating from electron propagation without scattering in the micron-scale^3^, considerable interest has been shown, especially towards high-speed graphene-based transistors, such as metal-oxide-graphene field-effect transistors (MOG-FET)^4^,^5^, graphene barristor^6^, and graphene-thin film semiconductor-metal tunnelling FET (GSM-TFET)^7^. These high-speed transistors can find applications in a variety of devices ranging from radio frequency (RF) switches and logic circuitry to photonic modulators^2^,^8^. To successfully incorporate graphene in these devices and to achieve excellent performance, the conformal and pinhole-free ultrathin film growth of high dielectric constant (high-*k*) materials on graphene with an excellent dielectric integrity is a fundamental technological requirement.

Atomic layer deposition (ALD), which is free of plasma damage and offers precise nanoscale thickness control with outstanding film quality and uniformity^9^, is considered to be the most promising technique for depositing high-*k* gate dielectrics on graphene. However, the crucial inherent limitation of ALD is that the uniform and high-quality film formation is determined by the condition of the exposed surface because the process kinetics is entirely based on the interaction and chemical adsorption of the precursors on the substrate surface^9^. In this respect, graphene is an inadequate nucleation template for enabling reaction with the ALD precursors because of its intrinsic lack of dangling bonds and functional groups on the exposed surface, which leads to a non-conformal growth of high-*k* dielectric films^10^,^11^. To overcome this challenge, several surface engineering techniques, such as surface functionalization (with nitrogen dioxide^12^, N_2_ plasma^13^, and ozone treatments^14^) and incorporation of seed layers (organic^11^,^15^,^16^ and metal (metal-oxide) layers^17^,^18^,^19^,^20^,^21^,^22^), have been suggested. These techniques have afforded the conformal and uniform deposition of various high-*k* dielectric films (mostly Al_2_O_3_ and HfO_2_), which are acceptable for device fabrication. However, to achieve high-performance graphene-based FETs, ultimately, the capacitance equivalent oxide thickness (CET) of a gate dielectric film needs to be downscaled using a HfO_2_ film with a higher *k* value than Al_2_O_3_, while maintaining a substantially low leakage current. In addition, for large-scale device integration, the CET downscaling is to be realised on graphene grown by chemical vapour deposition (CVD), which is most relevant to large-scale device fabrication.

Recently, we introduced GSM-TFETs as a novel graphene-semiconductor hybrid device for high- performance and low-power electronics^7^. The win-win strategy of using the graphene-semiconductor heterostructures was applied to selectively harness the high mobility resulting from the linear dispersion of graphene and the appropriate energy barrier of the semiconductor, which are two key parameters required for logic application. The inevitable power consumption increase associated with the continued miniaturization of complementary metal-oxide-semiconductor (CMOS) devices has become a serious issue. To reduce the power consumption or operating bias voltages, high-performance TFETs surpassing contemporary CMOS devices with subthreshold swing (SS) less than 60 mV/dec need to be developed. Instead of pursuing conventional Si or III-V TFETs requiring rigid substrates and with the external gate electric field perpendicular to the charge flow direction, we exploited vertical GSM-TFETs. Vertical GSMs exploit the external gate electric field parallel to the tunnelling charge carriers, leading to well-controlled transfer characteristics^7^.

In this paper, we report the scaling of the ALD HfO_2_ film thickness on CVD-grown monolayer graphene and demonstrate the fabrication of high-performance GSM-TFETs with a substantially small CET at the wafer scale. For this, several surface passivation methods, including N-methyl-2-pyrrolidone (NMP) treatment and the introduction of ultrathin sputtered ZnO and e-beam-evaporated Hf seed layers, were evaluated. Through a systematic comparison of the effects of these surface treatments on the subsequent growth of thermal ALD HfO_2_ films by various characterization techniques, we selected the most promising process of preparing high-quality HfO_2_ films on the monolayer graphene. Based on this screening process, we obtained scaled HfO_2_ films of excellent gate dielectric quality with a CET of ~1.5 nm in the MOG-FET structure. Finally, GSM-TFETs with the scaled HfO_2_ gate dielectric films showing SS values less than ~60 mV/dec were fabricated on an 8″ wafer.

## Results

### Sample Preparation and Structural Characterization of ALD HfO_2_ Films on Graphene

**Figure 1:**
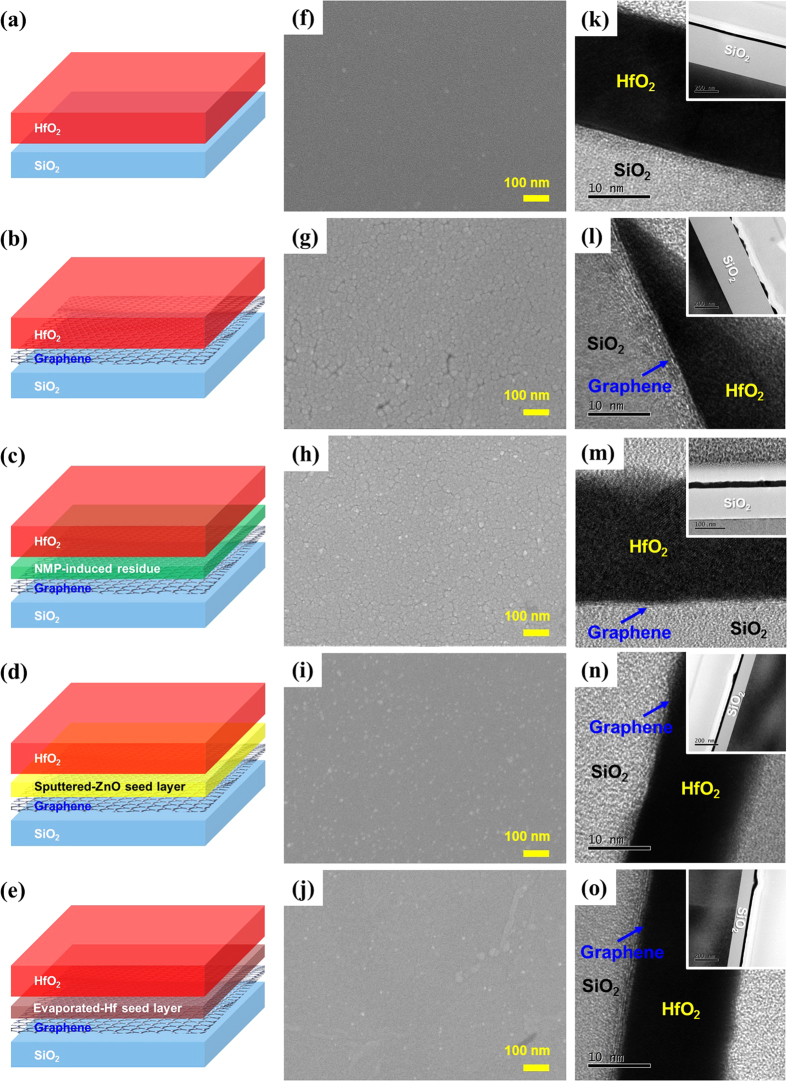
(**a–e**) Schematic diagrams, (**f–j**) plan-view SEM images, and (**k–o**) cross sectional TEM images of the samples characterized in this study. The film structures of the samples are (**a,f,k**) HfO_2_/SiO_2_, (**b,g,l**) HfO_2_/graphene/SiO_2_, (**c,h,m**) HfO_2_/NMP-treated graphene/SiO_2_, (**d,i,n**) HfO_2_/ZnO/graphene/SiO_2_, and (**e,j,o**) HfO_2_/Hf/graphene/SiO_2_. The insets of (**k–o**) are low magnification TEM images to show the global film morphology.

After the synthesis and transfer processes described in the experimental methods section, the monolayer graphene was chemically or physically treated to promote the prompt and uniform nucleation of successive ALD HfO_2_ films using three different methods i.e. surface treatment using NMP, introduction of ~3-nm-thick seed layers, including sputtered ZnO and e-beam-evaporated Hf films. Here, to demonstrate the influence of the organic solvent residue on graphene, NMP was used for the wet treatment, which was performed on a 2D MoS_2_ sheet with surface conditions (i.e. without dangling bonds and functional groups) similar to graphene^23^. Meanwhile, the ultrathin metallic Hf seed layer is expected to be converted to a Hf oxide film (or a Hf oxide film with a number of Hf-C bonds^18^,^22^) because it can be easily oxidized during sample preparation (because of unintended oxygen atoms in the e-beam evaporation chamber or air exposure during transfer) and also during the subsequent ALD step with the strongly oxidizing ambient^17^,^18^,^20^,^21^,^22^. The sample structures subjected to the three surface modification processes are schematically represented in Fig. 1(a,e); the two reference samples (without and with a monolayered pristine graphene on the SiO_2_/Si substrates) are also included. After preparing all the samples, the ALD was carried out using tetrakis-(ethylmethylamino) hafnium (TEMAHf) and H_2_O as precursors. We chose a relatively high deposition temperature of 200 °C, which is the lowest temperature within the stable ALD regime of HfO_2_ with a stable deposition rate (see Figure S1 in the Supporting Information) required to obtain a dielectric film with excellent quality. For samples without any surface modification and those subjected to only NMP treatment, the number of ALD cycles was adjusted to produce an ~20-nm-thick HfO_2_ film. This number was adjusted to produce ~17-nm-thick films for samples subjected to the two other surface modification procedures to produce the same total film thickness of 20 nm including the seed layer.

The surface morphology and cross sectional microstructure of the HfO_2_ films on graphene subjected to various chemical or physical surface modifications were examined using scanning electron microscopy (SEM) and transmission electron microscopy (TEM), respectively (the sample structures are shown on the right side of Fig. 1). The HfO_2_ film directly deposited on SiO_2_ without graphene showed a thickness of around 20 nm (as measured by TEM), and conformal deposition without pinholes was observed, as shown in Fig. 1(f,k). In contrast, when the HfO_2_ film was deposited on monolayered graphene transferred onto the SiO_2_ substrate, island growth behaviour appeared, as shown in Fig. 1(g,l). This is the typical morphology of ALD high-*k* films on CVD-grown graphene consisting of a small number of surface nucleation sites such as grain-boundaries, vacancies, and organic residues^13^,^22^. As shown in Fig. 1(h,m), the organic residues originating from NMP markedly improved the surface coverage of the ALD HfO_2_ film on graphene, suggesting that the surface properties of graphene may have been altered and a more facile nucleation of the subsequent HfO_2_ film may have been induced. However, the surface of the HfO_2_ film became rough with many pinholes (probably because of the unconnected boundaries between the islands), which may eventually provide high leakage current paths across the dielectric film.

The most conformal HfO_2_ films without boundaries and pinholes were achieved when either sputtered ZnO or e-beam-evaporated Hf served as the seed layer on graphene (see Fig. 1(i,n,j,o)). According to the plan-view SEM images, two samples (HfO_2_/ZnO/graphene and HfO_2_/Hf/graphene on SiO_2_) exhibited surface morphologies similar to that of the HfO_2_ film directly grown on the SiO_2_/Si substrate. HfO_2_ films with locally irregular topologies appeared randomly, which could possibly be attributed to the generation of process-induced nanoparticles on the graphene (see Figure S2); however, the topology might be improved by optimizing the seed-layer deposition conditions in future. The cross sectional TEM images of the two seeded samples shown in Fig. 1(n,o) reveal that the average thicknesses of all the deposited films (including the seed layers) are somewhat smaller than the expected value of 20 nm. The thickness values were ~17.1 nm and ~18.7 nm for the HfO_2_/ZnO/graphene and HfO_2_/Hf/graphene samples, respectively. This deviation may be attributed to a marginally longer incubation time for the early stages of the ALD on the ZnO- and Hf-seeded graphene surfaces than that on the SiO_2_ surface. Since ALD is a surface saturation-controlled process, the initial deposition rate is strongly dependent on the nature of the starting surface^9^. Furthermore, it is possible that the initial thicknesses of the ZnO and Hf films were smaller than the expected values because the deposition times were selected by extrapolating the deposition rates determined from the thicker films. We note that the surface roughness the HfO_2_ films on graphene subjected to various surface modifications was also examined using atomic force microscopy (AFM) (see Figure S3). The measured surface roughness with AFM agrees well with the results obtained from the SEM and TEM analyses.

For the phase identification and density measurement of the HfO_2_ films deposited on the graphene surface before and after applying the various surface modification processes, characterizations including X-ray diffractometry (XRD), medium energy ion scattering spectroscopy (MEIS), and X-ray reflectometry (XRR) were carried out. Figure S4 shows the XRD patterns of the ALD HfO_2_ films on different substrates. It is well known that ALD typically produces an amorphous HfO_2_ film on Si in the ALD regime and crystallization begins at temperatures >500 °C^24^. Consistent with this observation, all the ALD HfO_2_ films grown on the modified graphene surfaces were amorphous and did not show any diffraction peaks. To compare the quality of the ALD HfO_2_ films on the various modified graphene surfaces, MEIS was carried out and the spectra were used for simulations to estimate the HfO_2_ film density [*ρ*(HfO_2_)], as summarized in Table 1. The calculated density of the reference HfO_2_ film deposited on SiO_2_ was ~9.6 g/cm^3^, which is close to the value shown by bulk HfO_2_^25^. When the HfO_2_ film was deposited on pristine and NMP-treated graphene, the measured density decreased to ~8.7 g/cm^3^, presumably because of the incomplete HfO_2_ film growth, as shown by the SEM and TEM analyses (Fig. 1). On the other hand, the introduction of the ZnO and Hf seed layers on the graphene surface led to a nearly ideal HfO_2_ film density, reconfirming the conformal growth of the HfO_2_ film without pinholes.

### Integration Characterization of ALD HfO_2_ Films on Graphene

**Figure 2:**
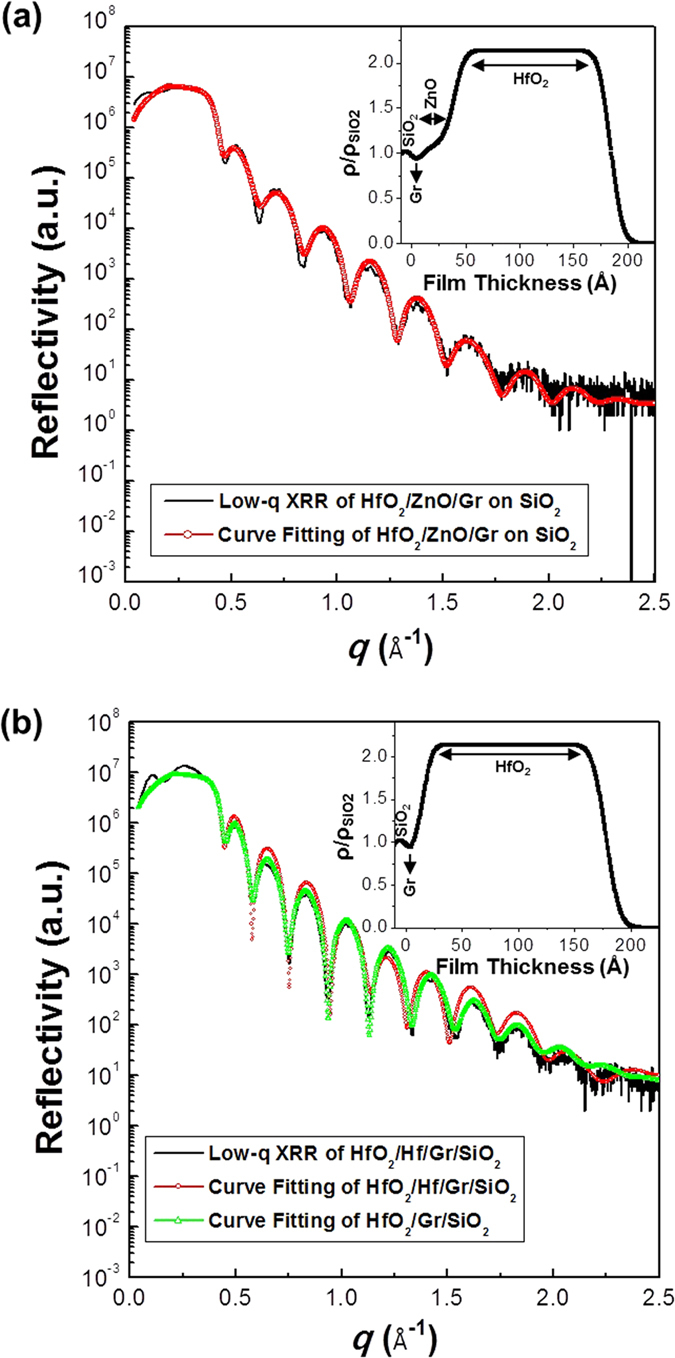
Measured and fitted XRR spectra of the HfO_2_/graphene/SiO_2_/Si structures with (**a**) ZnO and (**b**) Hf seed layers between HfO_2_ and graphene. For the Hf-seeded sample in (**b**), spectrum fitting was performed by assuming the seed layer as metallic Hf and HfO_2_. The insets of each figure represent the depth profiles of the electron density with respect to that of SiO_2_, which was extracted from the fitted curves. The inset figure of (**b**) was obtained from the best fitting result, assuming that the Hf seed layer is completely converted to HfO_2_. The simulated layer density and thickness values are also tabulated in Table 2.

To further compare the integrity of the HfO_2_ films and the embedded ZnO/Hf seed layers, low modulus of the momentum transfer (low-*q*^16^) XRR was carried out on the two samples (i.e. HfO_2_/ZnO/graphene and HfO_2_/Hf/graphene on SiO_2_) showing the most conformal HfO_2_ growth with a high film density (according to the MEIS analysis). Low-*q* XRR is a useful technique to estimate i) the layer thickness, ii) surface and interface roughness values, and iii) vertical surface density gradient and layer density of a multilayered structure^26^. The clear oscillatory behaviour observed in the measured XRR curves (Fig. 2) (termed as the Kiessig fringe), demonstrates that the HfO_2_ films were uniformly stacked on the graphene substrates with sharp interfaces. To obtain further details, least squares fitting of the reflectivity data was also carried out, as shown in Fig. 2. The insets of Fig. 2 show the electron density depth profiles derived from the least squares fitting. For the HfO_2_/ZnO/graphene/SiO_2_ sample, the estimated thickness of the HfO_2_/ZnO stacked layer was around 17.7 nm, including the 14.0-nm-thick HfO_2_ and 3.7-nm-thick ZnO layers (see Table 2), which agrees well with the results obtained from the cross sectional TEM (Fig. 1). The simulated thickness of the monolayered graphene buried under the sputtered-ZnO layer is ~0.3 nm, which is close to the theoretical value of 0.34 nm^27^. The roughness of graphene was much higher (*R*_*graphene*_ = ~2.5 nm) than that of the overlying ZnO layer (*R*_*ZnO*_ = ~0.76 nm). This increased interface roughness could be attributed to the mechanical deformation of graphene caused by the plasma-induced damage generated during the sputtering of the ZnO seed layer, which in turn formed a nearly uniform ZnO layer anchored on graphene. It is also possible that the ZnO/graphene nanocomposite layer leads to the requirement of a longer incubation time for the ALD of HfO_2_ because of the local appearance of an inert surface of graphene incorporated in the ZnO seed layer. The densities of each layer in the HfO_2_/ZnO/graphene/SiO_2_ structure obtained from the XRR measurements were *ρ*(HfO_2_) = 9.6 g/cm^3^, *ρ*(ZnO) = 4.6 g/cm^3^, *ρ*(graphene) = 2.2 g/cm^3^, and *ρ*(SiO_2_) = 2.7 g/cm^3^, which are in a reasonable agreement with the bulk values [*ρ*_o_(HfO_2_) = 9.68 g/cm^3^ 25, *ρ*_o_(ZnO) = 4.4 ~ 4.9 g/cm^3^ 28, *ρ*_o_(graphene/graphite) = 2.2 g/cm^3^ 29, and *ρ*_o_(SiO_2_) = 2.2 g/cm^3^ 25, respectively]. Furthermore, the XRR-derived HfO_2_ density matches well with the MEIS-derived value listed in Table 1. These results demonstrate that the ZnO seed layer in the HfO_2_/ZnO/graphene/SiO_2_ sample can be uniformly stacked as a separate layer forming a sharp interface with the overlying ALD HfO_2_ film; however, the underlying graphene layer is geometrically deformed (damaged) because of the subsequent ZnO sputtering.

Meanwhile, for the HfO_2_/Hf/graphene/SiO_2_ sample, the least squares fitting was performed by assuming the seed layer as either a metallic Hf layer (fitting curve in red) or a HfO_2_ layer (fitting curve in green), as shown in Fig. 2(b). When compared with the measured reflectivity data, the best fitting was obtained when the seed layer was assumed to be HfO_2_, which verifies that the deposited Hf seed layer was mostly converted to HfO_2_. The thickness, density, and roughness values of the HfO_2_ and graphene layers were extracted from the best fit curve and are summarized in Table 2. Despite the identification of the delayed growth of the ALD HfO_2_ film (similar to the ZnO-seeded sample and also expected from the TEM analysis shown in Fig. 1), most of the extracted parameters were close to the values expected from the HfO_2_/ZnO/graphene/SiO_2_ sample. In contrast to the HfO_2_/ZnO/graphene/SiO_2_ sample, the roughness values of both the HfO_2_ film and the graphene layer were quite low, implying that the physical damage on the underlying graphene is quite minimal during the subsequent e-beam evaporation of the Hf seed layer.

### Interfacial Characterization of the ALD HfO_2_ Films on Graphene

**Figure 3:**
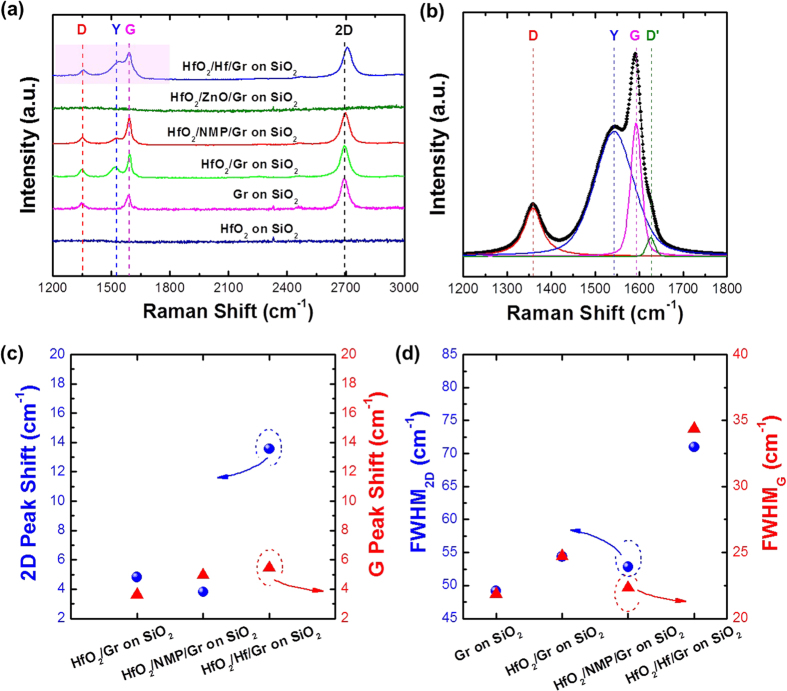
(**a**) Raman spectra collected from the ALD HfO_2_ samples on monolayered graphene transferred on to SiO_2_/Si substrates. Prior to the ALD of HfO_2_, the monolayered graphene was pretreated using various methods. For reference, the Raman spectrum of the HfO_2_/SiO_2_/Si sample without graphene is also included. (**b**) Enlarged spectrum of the HfO_2_/Hf/graphene/SiO_2_/Si sample with deconvoluted peaks showing the Y and D′ peaks. (**c**) The 2D and G peak shifts after the HfO_2_ deposition on the graphene layers subjected to various surface treatments. (**d**) FWHM values of 2D and G Raman features for the various samples.

Raman spectroscopy is one of the most widely used techniques for assessing the quality of graphene and its possible interaction with adjacent layers. In this study, we carried out Raman spectroscopy on the various samples at room temperature to analyse the effects of surface passivation and the subsequent ALD of HfO_2_ on the integrity of graphene (Fig. 3(a)). The main features in the Raman spectra of the pristine graphene transferred to a SiO_2_/Si substrate include the D (~1350.4 cm^−1^), G (~1587.8 cm^−1^), and 2D (~2694.6 cm^−1^) peaks. Both the shape of the 2D peak and its higher intensity (*I*_*2D*_) than the G peak (*I*_*G*_) with a low intensity D peak confirm that the synthesised graphene is a monolayer and is of high quality^30^. When the ALD HfO_2_ films were deposited on the pristine, NMP-treated, and Hf-seeded graphene monolayers, the Raman spectra were quite similar to that of the sample without any surface treatment, except for the appearance of additional peak features as shoulders on the G peak [denoted as Y and D′ peaks in Fig. 3(a,b)]. The possible origins of the appearance of the Y and D′ peaks will be discussed below. As stated above, nearly complete coverage of the subsequently deposited HfO_2_ film was only achieved by the introduction of the ZnO and Hf seed layers. However, in the case of the ZnO-seeded sample, all the graphene-related Raman features were completely removed, as shown in Fig. 3(a). This implies that the graphene layer was severely damaged during the ZnO seed layer formation, probably because of the sputtering-induced plasma damage, which also supports the increase in roughness observed by the XRR analysis. In contrast, when e-beam-evaporation was used to deposit the Hf seed layer, such damages could be avoided and the distinct intrinsic graphene peaks were retained, as shown in Fig. 3(a) (first spectrum from the top).

The following additional observations were made from the Raman spectra, leading to information on the interface between the HfO_2_/Hf seed layer and the graphene layer. First, a D′ peak (a red-shifted additional feature) and Y peak (a blue-shifted additional feature) appear as shoulders on the G peak, as shown in Fig. 3(b). In addition, the 2D peak showed a significant shift (>13.5 cm^−1^) and widening (>22 cm^−1^) in comparison to the peak shown by pristine graphene on SiO_2_, as shown in Fig. 3(c,d). The D′ peak is attributed to the phonon-induced intraband electronic transitions, which is observed when graphene is doped with metals^31^. Therefore, the appearance of the low-intensity D′ peak in the Raman spectra confirms the occurrence of a slight charge transfer between the HfO_2_/Hf-seed layer and the graphene layer. The 2D peak shift and widening can be attributed to either carrier density modulation induced by a charge transfer or the introduction of mechanical strain by an additional seed layer^31^. When the 2D peak shift (Δ*P*_*2D*_) is larger than the G peak shift (Δ*P*_*G*_), the strain effect is dominant^31^. In contrast, when Δ*P*_*2D*_ < Δ*P*_*G*_, the charge carrier density modulation effect is the main factor causing the peak shift^31^. In our case, Δ*P*_*2D*_ (~13.5 cm^−1^) is much larger than Δ*P*_*G*_ (~5.5 cm^−1^), suggesting a strong strain effect caused by the adjacent layers, specifically the Hf seed layer (see Figure S5 in the Supporting Information). Note that the peak shift caused by any heating effects (Δ*P*_*heating*_ < 2 cm^−1^) is much smaller than the shifts observed in this study^32^.

Further, as mentioned previously, with the exception of the ZnO-seeded sample, which resulted in the complete removal of all the Raman peaks, we observed the appearance of a Y peak between the D and G peaks only when HfO_2_ was covered on the graphene surface. The Y peak was absent in the Raman spectra obtained from HfO_2_ on SiO_2_ without graphene (the bottom-most in Fig. 3(a)). Also, the intensity of this peak was the highest when the coverage of HfO_2_ on graphene reached a maximum (Hf-seeded sample); therefore, we can postulate that this feature is closely related to the amount of interface between HfO_2_ and graphene and probably, the number of bonds between Hf and graphene (observed as a metal-graphene bond formation in^31^). However, the appearance of the Y peak (Hf-graphene bonds) does not produce any change in the D peak shape and intensity, which suggests that it does not generate the structural defects in graphene. For the NMP-treated sample, although a better HfO_2_ coverage was obtained than that observed in the HfO_2_/pristine graphene sample, the Y peak intensity was lower, which contradicts our postulate. However, the lower Y peak intensity may be attributed to the localized formation of the Hf-graphene bonds via the pinholes if the HfO_2_ islands grow preferentially on the organic residues in the NMP-treated sample, as discussed previously in the context of SEM analysis (Fig. 1(h)). Figure 3(d) plots the change in the full width at half maximum (FWHM) of the 2D and G peaks for samples subjected to the different graphene-passivation methods. Overall, the increase in the HfO_2_ coverage, i.e. the increase in the number of Hf-graphene bonds at the interface resulted in the widening of the 2D and G peaks, probably because of the interrupted Raman scattering from graphene^33^. Only the NMP-treated sample was an exception and showed a much smaller change because of the lower areal density of the Hf-graphene bonds at the interface, as discussed above.

### Downscaling and Electrical Evaluation of the ALD HfO_2_ Films on Graphene

**Figure 4:**
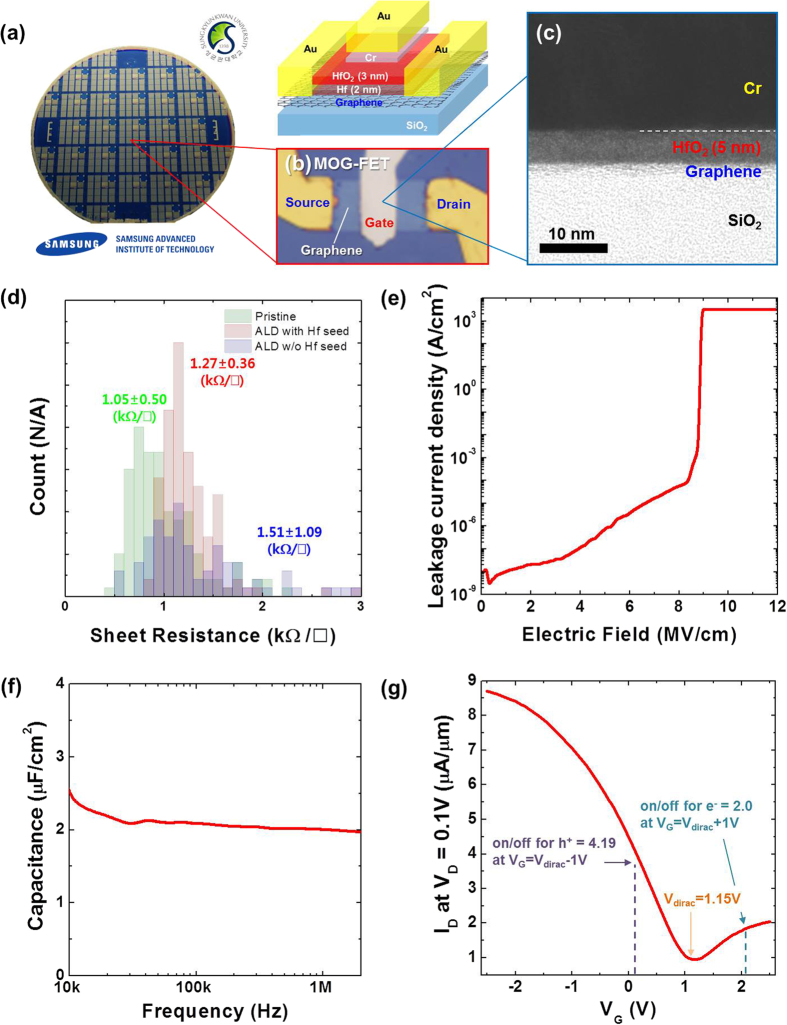
(**a**) Optical image of the MOG-FET arrays fabricated on a 6″ Si wafer and a schematic illustration showing the structure of the MOG-FET device. (**b**) Optical microscope image of a fabricated MOG-FET unit device. (**c**) Cross sectional TEM image showing the HfO_2_ gate dielectric layer with a thickness of ~5 nm (including the seed layer converted to a HfO_2_ layer) on monolayered graphene. (**d**) Statistical distribution of the sheet resistance of a monolayered graphene before and after the ALD of HfO_2_ with and without an e-beam-evaporated Hf seed layer. Representative electrical characteristics measured from the fabricated MOG-FET devices: (**e**) gate dielectric leakage current, (**f**) gate capacitance as a function of the frequency, and (**g**) transfer curve (*I*_D_-*V*_G_).

In summary, analyses of the HfO_2_ coverage and graphene integrity on the various surface-passivated graphene surfaces confirmed that the introduction of the e-beam-evaporated Hf seed layer is the best process to obtain amorphous HfO_2_ films with uniform coverage, while retaining the graphene integrity. Subsequently, using the Hf-seeding method, we fabricated an array of MOG-FETs with the monolayered graphene at a wafer scale and decreased the HfO_2_ thickness to ~5 nm (including the oxidized Hf seed layer). The detailed fabrication process steps are described in the experimental methods section. As illustrated in Fig. 4(a,b), the MOG-FET devices were built on a 6″ SiO_2_/Si wafer in a top-gated geometry and the gate length and channel width were fixed at 5 μm and 10 μm, respectively. The TEM analysis of the cross section of the FET sample prepared via focused ion beam milling indicated the presence of an ~5-nm-thick gate dielectric (HfO_2_) layer between the graphene and gate metal stack, as shown in Fig. 4(c).

Figure 4(d) shows the change in the statistical distribution of the sheet resistance of the transferred graphene after the ALD of HfO_2_ with and without the Hf seed layer. The as-transferred pristine graphene showed an average sheet resistance of ~1.05 kΩ/□. After the ALD of HfO_2_ directly on the transferred graphene, the value increased to ~1.51 kΩ/□ and the standard deviation almost doubled. We are unable to provide a definite explanation for this at the moment; however, we speculate that this observation can be attributed to the direct exposure of the monolayered graphene to the highly oxidizing ambient encountered in the ALD. On the contrary, when the graphene layer was pre-coated with the e-beam evaporated Hf seed layer, the increase in the sheet resistance of graphene was much smaller (with the value reaching only ~1.27 kΩ/□) with a similar standard deviation even after the ALD of HfO_2_. This indicates that the introduction of the ultrathin Hf seed layer is also beneficial towards maintaining the intrinsic quality of the graphene. We note that the MOG FETs built on SiO_2_ (100 nm)/Si in back-gated configuration before and after Hf deposition were also measured (see Figure S6). The measured MOG FET performance agrees well with the results obtained from the sheet resistance of the graphene of Fig. 2(d).

The dielectric quality of the formed ~5-nm-thick HfO_2_ film was evaluated, as shown in Fig. 4(e,f). The leakage current was similar to that observed in ALD HfO_2_ on metal and Si wafer structures of similar thicknesses (Ti/Au/HfO_2_/Cr/Au and Ti/Au/HfO_2_/Si, as shown in Figure S7 (a) and (b), respectively). The film also showed a reasonably high hard breakdown field of ~9 MV/cm [see Fig. 4(e)]. The gate capacitance was measured at alternating current (AC) frequencies ranging from 10 kHz to 2 MHz (Fig. 4(f)), which confirms that the CET of the HfO_2_ gate dielectric is around 1.5 nm. In terms of the FET performance, the representative drain current (*I*_*D*_) versus gate bias (*V*_*G*_) curve is shown in Fig. 4(g), which indicates a Dirac voltage of ~1.15 V. The on/off ratio values of ~2.0 and ~4.19 were obtained for electrons and holes, respectively, when the gate voltages shifted from the Dirac voltage by ±1 V. All these results are consistent with the performance expected from high-quality ultrathin high-*k* dielectrics on monolayered graphene, which can be used as a suitable building block for the fabrication of high-performance graphene tunnelling transistors.

### Fabrication of High-performance Graphene Tunnelling Transistors

**Figure 5:**
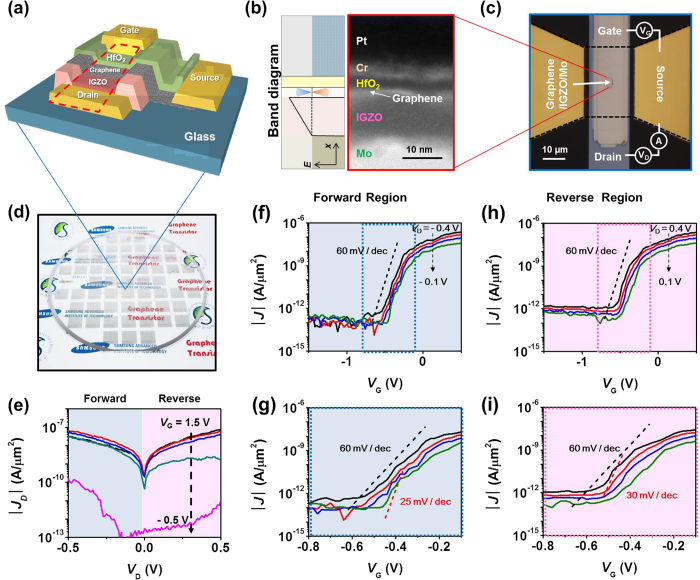
(**a**) Schematic illustration showing the structure of a GSM-TFET. (**b**) Schematic band diagram and the corresponding cross sectional TEM image of the GSM tunnelling junctions. (**c,d**) Optical images of the GSM-TFET and an 8″ glass wafer with the integrated GSM TFETs, respectively. (**e**) *J*_D_-*V*_D_ characteristics at various *V*_G_ values. (**f,g**) *J*_D_-*V*_G_ characteristics at various *V*_D_ values under forward bias shown in (**e**) and the enlarged view of the dotted box in (**f**), respectively. (**h,i**) *J*_D_-*V*_G_ characteristics at various *V*_D_ values under reverse bias shown in (**e**) and the enlarged view of the dotted box shown in (**h**), respectively.

We integrated the highly scaled HfO_2_ gate dielectric film into GSM-TFETs on an 8″ glass wafer. The GSM-TFET consists of vertical tunnelling junctions with graphene, InGaZnO (IGZO), and Mo electrode functioning as the work function tunable source, tunnelling barrier, and drain electrode, respectively, as shown in Fig. 5(a). Previously, by tailoring the barrier height and thickness of the built-in triangular barrier enabling Fowler-Nordheim tunnelling at low source-drain bias voltages, low-voltage operating GSM-TFETs with an on/off ratio of 10^6^ were achieved at drain voltage (*V*_D_) = 0.5 V^7^. However, for low-power operation at an integrated circuit level, in addition to *V*_D_, *V*_G_ needs to be downscaled. Figure 5(b–d) show the band diagram of a GSM-TFET with the corresponding cross sectional TEM image and optical microscopy images of the unit/integrated GSM-TFETs on an 8″ glass wafer. The TEM image (Fig. 5(b)) shows that the ~5-nm-thick HfO_2_ film, which controls the work function of graphene, is well defined between the gate electrode and graphene. The drain current density (*J*_D_) versus *V*_D_ characteristics (Fig. 5(e)) show an Ohmic-like behaviour at *V*_G_ > 0.5 V because of the increased charge injection through the tunnel barrier with a high asymmetricity, while an abrupt drop of *J*_D_ is observed at *V*_G_ < 0.5 V. The designed triangular barrier causes current rectification because in the forward direction *V*_D_ compensates for the built-in barrier to allow for a relatively enhanced current flow; in our case, the negative *V*_D_ appeared to be in the forward direction. From the observed asymmetricity, we can determine that the energy barrier is higher at the graphene/IGZO interface than between the IGZO/Mo interface. Hence, in addition to the nearly Ohmic contact at the IGZO/Mo interface by adopting a Mo electrode in the GSM-TFETs^34^,^35^,^36^, the energy barrier between the graphene and the semiconductor leads to a modulation of the barrier height by tuning graphene work function with a highly scaled gate oxide film, resulting in a high on/off ratio. In the GSM-TFET, the energy barrier control between the graphene and the semiconductor is more desirable than the fixed barrier between the semiconductor and metal because the gate modulation of graphene work function can change the tunnelling probability more effectively, as explained below.

Based on the discussion in our previous report^7^, the n-type operation of the graphene–IGZO–Mo devices (see Fig. 5(f–i)) can be explained qualitatively, as follows. As *V*_G_ is varied from negative to positive, the effective electric field between graphene and the metal changes in accordance with the lowered graphene work function and band bending in the semiconductor^37^ and simultaneously, the energy barrier between graphene and IGZO is also lowered, resulting in an increased electron injection leading to the on-state. To understand the tunnelling behaviour in our device, we consider the tunnelling probability between the graphene and the metal (Mo) through the barrier (IGZO)^7^: 
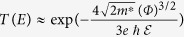
, where m^*^ is the effective mass of an electron in the barrier, *Φ* is the energy barrier between graphene and the drain electrode, *e* is the electric charge, 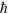 is the reduced Planck constant, and ε is the electric field across the barrier. As *V*_G_ is varied, *T*(*E*) is modulated exponentially by the change in 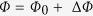 and 
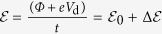
, where *t* is the thickness of the barrier and 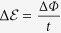 because of the increased or decreased graphene work function (Δ*Φ*). Therefore, a high on/off ratio is achieved by the modulation of the electric field across the junctions, and more specifically, by the reduced barrier height.

Figure 5(f,g) show the *J*_*D*_ versus *V*_G_ characteristics for various *V*_D_ values at forward bias. We obtained an average SS value of 60 mV/dec for up to four orders of magnitude of current modulation ranging from *J*_*D*_ = 10^−13 ^A/μm^2^ to 10^−9 ^A/μm^2^, and a minimum swing of 25 mV/dec was obtained for one order of magnitude of current modulation ranging from 10^−13 ^A/μm^2^ to 10^−12 ^A/μm^2^ at |*V*_D_| < 0.5 V. The threshold *V*_G_ ranged from −0.55 V to −0.45 V as *V*_D_ was varied from −0.4 V to −0.1 V. In forward bias, the observation of a shifted *V*_G_ is natural because each *V*_D_ compensates for the built-in potential corresponding to a different energy barrier such that for an increased *V*_D_, the threshold voltage appears at a lower *V*_G_. Therefore, the work function of graphene should be increased to turn the device off. Nevertheless, a value of 25 mV/dec is a record-low value among field-effect devices consisting of graphene or transition metal dichalcogenides, and their heterostructures and the less-than-60 mV/dec value directly proves the carefully designed tunnelling barrier and its successful operation. On the other hand, under reverse bias, we observed an average SS of 60 mV/dec for up to three orders of magnitude of current modulation ranging from *J* = 10^−12 ^A/μm^2^ to 10^−9 ^A/μm^2^ and a minimum swing of 30 mV/dec for one order of magnitude of current modulation ranging from 7 × 10^−12 ^A/μm^2^ to 7 × 10^−11 ^A/μm^2^ at *V*_D_ < 0.5 V. In reverse bias, the threshold *V*_G_ change with various *V*_D_ is comparable to that in forward bias. Note that both in forward and reverse biases, *V*_D_ and *V*_G_ can be less than 0.5 V, which can enable low-power consumption in logic circuitry. Despite the relatively low on-current of 3 × 10^−7 ^A/μm^2^, this is the first proof-of-concept demonstration of a device with 2D materials showing SS values <60 mV/dec and there is much room for further improvement. First, the device fabricated here has a relatively large tunnelling thickness of 20 nm compared to 5–10 nm used for most TFETs and the current can be expected to exponentially enhance with further thickness scaling. Moreover, thorough interface control between each layer at the vertical tunnel junction can be applied to the fabrication process (see Figure S8 for the measured hysteresis behaviour of the GSM TFETs obtained by sweeping the gate voltage), for example, by curing defect sites in IGZO through moderate annealing^34^,^38^,^39^ and by adopting clean graphene transfer techniques^40^,^41^.

## Discussion

In this work, ALD HfO_2_ films were grown at 200 °C on CVD graphene monolayers after various surface passivation protocols, such as NMP treatment, sputtering of a ZnO film, and e-beam-evaporation of a Hf film. NMP treatment significantly enhanced the HfO_2_ film coverage by the generation of organic residues although a low film density with many pinholes was obtained. The highest surface coverage and an ALD HfO_2_ film of nearly ideal density could be achieved by the introduction of ZnO or Hf seed layers. The sputtering of the ZnO seed layer severely damaged the graphene; however, the e-beam-evaporated Hf seed layer, which was probably converted to Hf oxide during the subsequent ALD process, provided the best integration template with graphene in the absence of any physical damage. With the Hf seeding by e-beam evaporation, the thickness of the HfO_2_ gate dielectric was scaled down to ~5 nm and the functioning of top-gated MOG-FETs with monolayered graphene was successfully demonstrated on a 6″ wafer. Further, an excellent HfO_2_ gate dielectric quality with a high breakdown field of ~9 MV/cm and reasonably low leakage current was achieved at a CET of ~1.5 nm. Finally, graphene heterojunction tunnelling transistors with an SS of <60 mV/dec were successfully fabricated on an 8″ glass wafer. The high-performance and low operating voltage of our device stemming from the high quality, pinhole-free, and uniform deposition of an ultrathin ALD HfO_2_ dielectric on monolayered graphene in the wafer scale are anticipated to provide a versatile opportunity as a scale-up approach to commercialize graphene-based technologies. Furthermore, we have proved tunnelling operation surpassing the thermal limit of 60 meV/dec in this study, which is particularly important because the tunnelling device exploiting graphene can also be applied to very large scale thin-film transistors for display and transparent/flexible electronics.

## Methods

### Synthesis and Transfer of Graphene

Monolayered graphene was synthesized on copper (Cu)-evaporated 6″ Si wafer by CVD using hydrogen and methane. The sample was spin-coated with poly(methyl methacrylate) (PMMA) and then soft-baked to improve the adhesion of PMMA to graphene. Then, the Cu film was peeled off from the Si wafer and completely etched away in a Cu etchant. Finally, the separated graphene layer was transferred onto a 6″ Si wafer covered with a thermally grown SiO_2_ film (300 nm in thickness).

### Surface Passivation and ALD of HfO_2_ on Graphene

For the NMP treatment, the graphene-transferred wafer was soaked in NMP for 60 min and then blow-dried with high-purity N_2_ gas. The thickness of the two seed layers was fixed at approximately 3 nm. The ZnO seed layer was sputtered at an RF power of 100 W, while the Hf seed layer was e-beam-evaporated. After passivating the graphene surface, ALD of HfO_2_ was carried out at 200 °C by separately injecting TEMAHf and H_2_O vapour with N_2_ purging steps in-between. The deposition rate of the HfO_2_ films on a SiO_2_ (300 nm)/Si substrate was monitored to be roughly 0.07 nm/cycle (Figure S1). The accurate ALD deposition rate of HfO_2_ on the various functionalized graphene surfaces was unavailable; therefore, the number of deposition cycles was chosen to produce a total film thickness of ~20 nm, including the seed layer, based on the deposition rate on SiO_2_.

### MOG-FET Fabrication

For the fabrication of the top-gated FET devices using monolayered graphene at the wafer scale, a graphene monolayer was grown on a 6″ Cu/SiO_2_/Si wafer by CVD. Then, the graphene film was transferred on the 6″ 100 nm SiO_2_/n^++^ Si wafer by the transfer process described above. The graphene channel was defined by photolithography and O_2_ reactive ion etching with a gold hard mask. The source/drain electrodes (100 nm Au/10 nm Ti) were patterned by photolithography and lift-off after deposition by e-beam evaporation. As the gate dielectric, ALD HfO_2_ was introduced on the e-beam evaporated Hf seed layer, which yielded a total dielectric (HfO_2_) thickness of ~5 nm including the transformed HfO_2_ seed layer formed during the ALD. Finally, a 100 nm Au/10 nm Cr top gate electrode was deposited and defined in a manner similar to the source/drain electrode formation. Metal pads (100 nm Au/10 nm Ti) were subsequently fabricated on each metal electrode for stable electrical measurement.

### GSM-TFET Fabrication

The GSM-TFETs were fabricated on an 8″ glass wafer using standard semiconductor processes. First, the sputtered drain electrode (Mo, ~200 nm in thickness) was defined by photolithography and dry etching. To deposit the amorphous IGZO thin film (10–20 nm in thickness), a target prepared by mixing GaO, InO, and ZnO powders was sputtered by RF plasma using Ar/O_2_. The IGZO thin film was patterned using dilute HF etchant. A 100-nm-thick SiO_2_ layer was deposited by plasma-enhanced CVD at 200 °C and patterned by dry etching. The wafer-scale CVD graphene was then transferred to form the graphene–IGZO–metal junctions and patterned by O_2_ plasma. The graphene was then placed in contact with the source electrode (Au, 100-nm-thick). Finally, after forming the gate dielectric HfO_2_ (5–6 nm in thickness) on graphene, the gate electrode (Pt/Cr, 45/5 nm) was stacked to complete the device fabrication.

### Film and FET Characterization

The growth behaviour of the ALD HfO_2_ film was examined both by field-emission SEM (JSM 7000F, JEOL) and TEM (Tecnai Osiris, FEI). The HfO_2_ films were characterized by XRD (D8 Advance, Bruker; *λ* = 1.78897 nm, at 40 kV and 100 mA) and XRR (X’PERT-PRO MRD, Panalytical; employing a ceramic X-ray tube (*λ* = 0.154 nm) and a high-resolution goniometer (resolution = ± 0.0001°)). The graphene layers in the samples were also characterized by a Renishaw micro-Raman spectroscopy with an excitation wavelength of 514 nm. The electrical characteristics of the fabricated FET devices were measured using a Keithley 4200-SCS semiconductor parameter analyser in a N_2_ chamber probe station.

## Additional Information

**How to cite this article**: Jeong, S.-J. *et al.* Thickness scaling of atomic-layer-deposited HfO_2_ films and their application to wafer-scale graphene tunnelling transistors. *Sci. Rep.* **6**, 20907; doi: 10.1038/srep20907 (2016).

## Supplementary Material

Supplementary Information

## Figures and Tables

**Table 1:** Densities of the HfO_2_ films grown on various substrates extracted from the MEIS measurements.

	HfO_2_ on SiO_2_	HfO_2_ on graphene	HfO_2_ on NMP-treated graphene	HfO_2_ on ZnO- seeded graphene	HfO_2_ on Hf-seeded graphene
**Density (g/cm**^**3**^)	9.6	8.7	8.7	9.6	9.6

The density of bulk HfO_2_ is ~9.68 g/cm^2^ 25.

**Table 2:** Density, thickness, and roughness values of each layer in the HfO_2_ on ZnO and Hf-seeded samples extracted from the XRR measurements.

	Thickness (nm)	Density (g/cm^3^)	Roughness (nm)
**SiO**_**2**_	–	2.7	–
**Graphene**	0.3	2.2	2.50
**ZnO**	3.7	4.6	0.76
**HfO**_**2**_	14.0	9.6	0.88
**SiO**_**2**_	–	2.5	–
**Graphene**	0.3	2.1	0.81
**HfO**_**2**_	18.8	9.6	0.88

In the case of the Hf-seeded sample, the seed layer is assumed to be completely converted to HfO_2_.
