# Supplementary material for: Thickness scaling of atomic-layer-deposited HfO2 films and their application to wafer-scale graphene tunnelling transistors
Source: Sci Rep. 2016 Feb 10;6:20907. doi: 10.1038/srep20907 (PMC4748263; doi:10.1038/srep20907)
Supplement: Supplementary Information [file srep20907-s1.pdf]

SUPPLEMENTARY INFORMATION

Thickness scaling of atomic-layer-deposited HfO_2 films and their application to wafer-scale graphene tunnelling transistors

Seong-Jun Jeong[1†], Yeahyun Gu[2†], Jinseong Heo[1†], Jaehyun Yang[2], Chang-Seok Lee[1], Min-Hyun Lee[1], Yunseong Lee[1], Hyoungsub Kim[2*], Seongjun Park[1*], Sungwoo Hwang[1]

[1]Device Laboratory, Device and System Research Center, Samsung Advanced Institute of Technology, Suwon 443-803, Korea. [2]School of Advanced Materials Science and Engineering, Samsung-SKKU Graphene/2D Center, Sungkyunkwan University, Suwon 440-746, Korea.

†These authors contributed equally to this work.

Correspondence and requests for materials should be addressed to H. K. (hsubkim@skku.edu) or S. P. (s3.park@samsung.com).

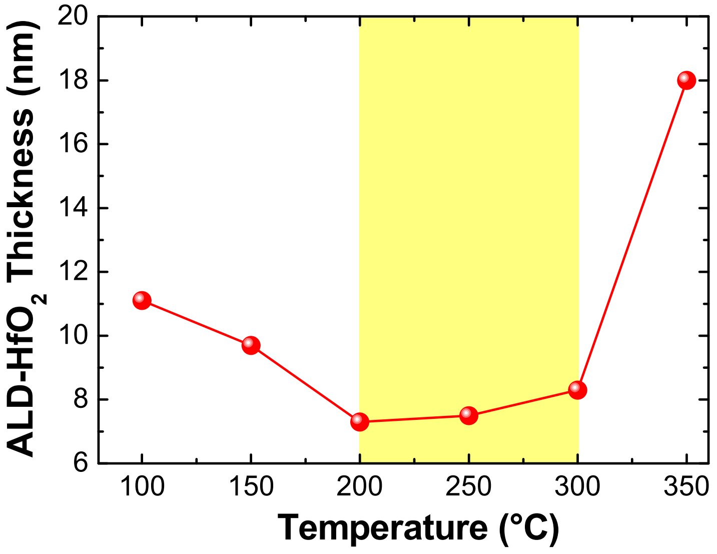

Figure S1. Thickness of the ALD HfO_2 films deposited on SiO_2 (300 nm)/Si substrates as a function of the deposition temperature. The total number of deposition cycles was fixed at 100. The thickness was measured using a spectroscopic ellipsometer (SE-MG1000, Nanoview). The ALD process window with a steady deposition rate was chosen to at the temperature ranging between 200 and 300 °C.

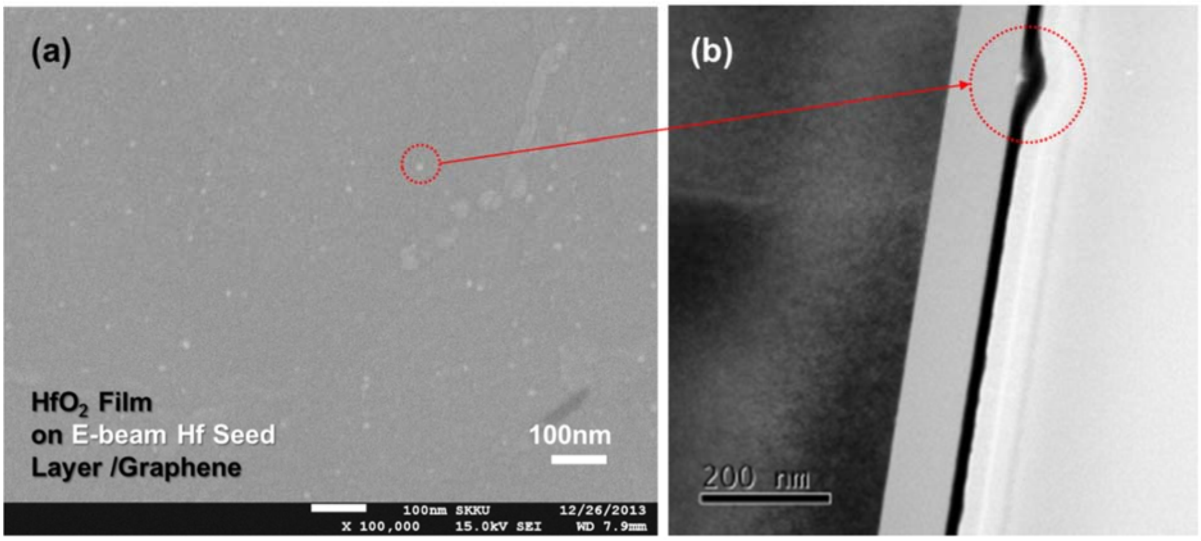

Figure S2. (a) Plan-view SEM and **(b)** cross sectional TEM images of the HfO_2/Hf/graphene/SiO_2 on Si sample. The red-dotted circle shows the sporadically observed irregular topology of the HfO_2 film, probably induced by the accidental generation of nanoparticles on graphene during the e-beam evaporation of the Hf seed layer.

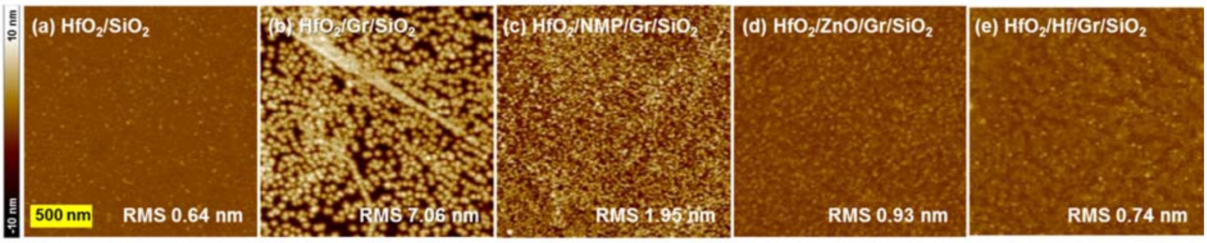

Figure S3. (a-e) Topographic AFM images of the samples characterized in this study. The film structures of the samples are **(a)** HfO_2/SiO_2, **(b)** HfO_2/graphene/SiO_2, **(c)** HfO_2/NMP-treated graphene/SiO_2, **(d)** HfO_2/ZnO/graphene/SiO_2, and **(e)** HfO_2/Hf/graphene/SiO_2. The measured RMS values are also included in each figure.

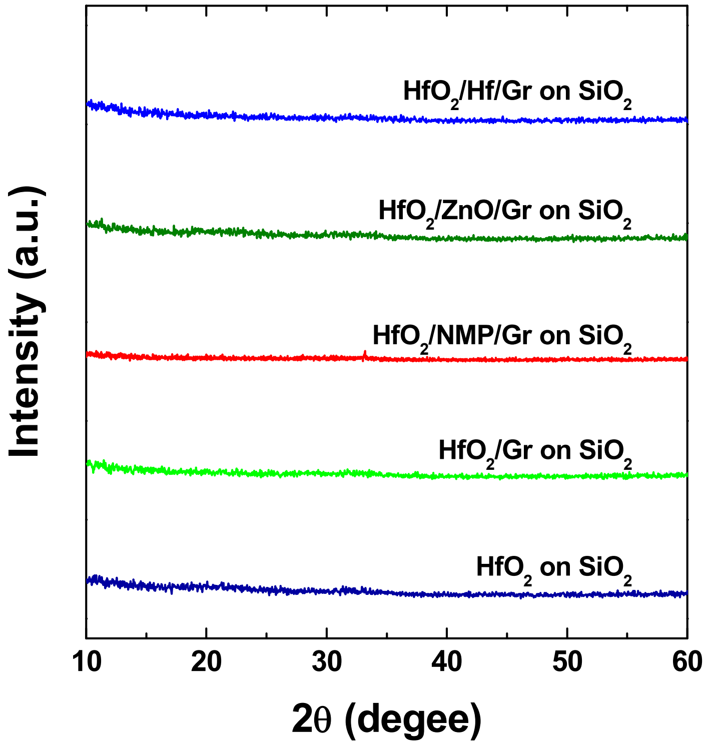

Figure S4. XRD patterns (θ-2θ) obtained from the ALD HfO_2 samples on monolayered graphene subjected to surface treatment by various methods and transferred on to SiO_2/Si substrates. The figure also includes two reference samples with and without pristine graphene.

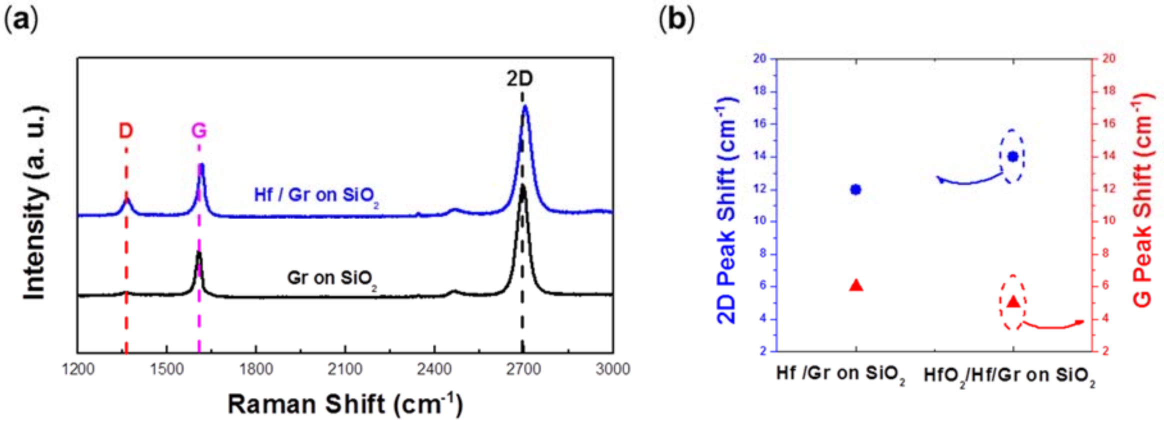

Figure S5. (a) Raman spectrum of the graphene/SiO_2 samples with and without the Hf seed layer. (b) The 2D and G peak shifts of the Hf/graphene/SiO_2 and ALD-HfO_2/Hf/graphene/SiO_2 samples. $\varDelta P_{2D(Hf/Gr)}$ was slightly larger than $\varDelta P_{G(Hf/Gr)}$. Raman peak shift values of the Hf/Gr sample confirmed the Hf metal effect on graphene surface.

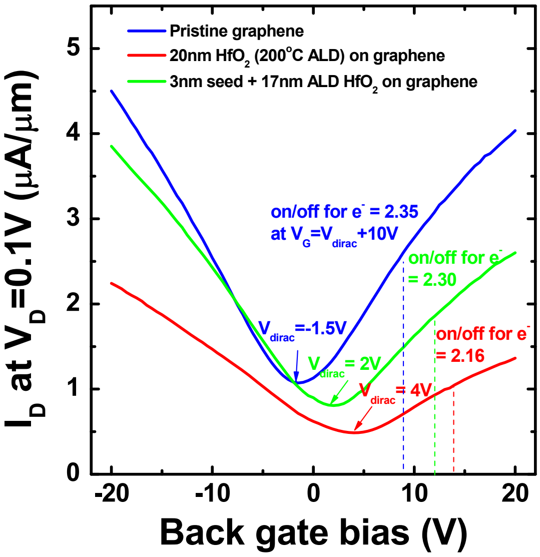

Figure S6. Representative transfer curves (I_D-V_{BG}) measured from the MOG FET devices with back-gated configuration. For the FET with pristine graphene, the on/off ratio value of 2.35 was obtained for electrons, when the gate voltages shifted from the Dirac voltage by +10 V. After the e-beam evaporation of the Hf seed layer on the graphene, the on/off ratio value was 2.30, which exhibited almost a negligible change. On the other hand, after the direct ALD of HfO_2 on graphene, the value was 2.16, which was relatively low. As mentioned in the sheet resistance of the graphene of Figure 2(d), the intrinsic quality of the graphene remains intact even after the introduction of the ultrathin Hf seed layer. Thus, we have not found a critical influence of the Hf seed layer on the device performance.

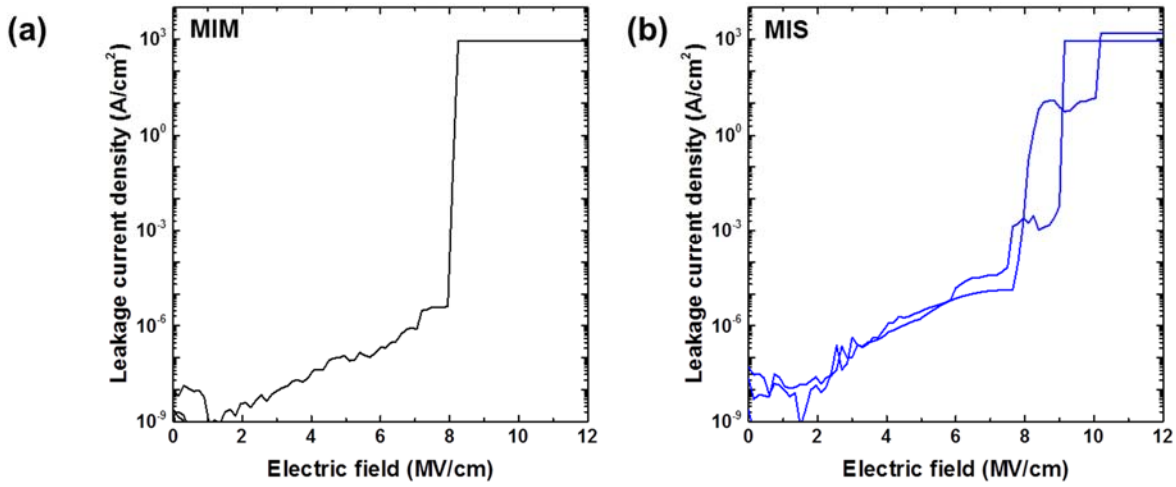

Figure S7. Leakage current characteristics of the ~5-nm-thick ALD HfO_2 film with an e-beam-evaporated Hf seed layer measured from the **(a)** metal-insulator-metal (Ti/Au/HfO_2/Cr/Au) and **(b)** metal-insulator-semiconductor (Ti/Au/HfO_2/Si) structures.

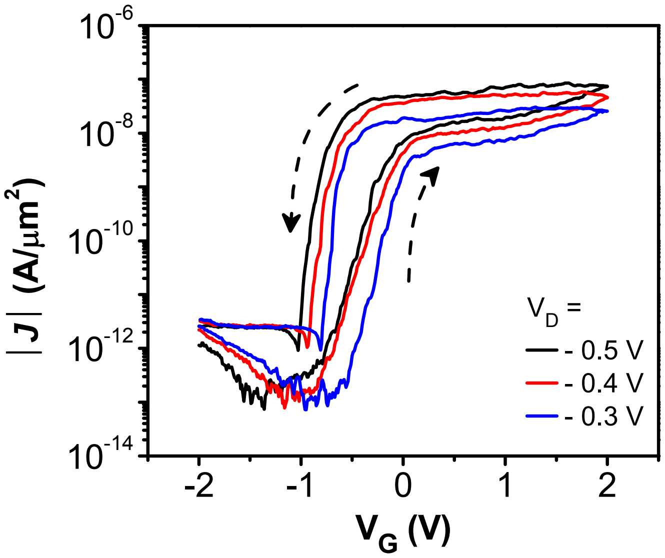

Figure S8. Hysteresis behaviour of the GSM TFETs obtained by sweeping the gate voltage. The results may be caused by the surface dipoles originated from accidentally-introduced nanoparticles on graphene during the e-beam evaporation of the Hf seed layer and also from tape residues/interface-trapped water molecules introduced during the graphene transfer process. The threshold voltage shift was around 0.2 V regardless of V_D. We also found that SS was degraded from 50-60 mV/dec to 140-150 mV/dec. This can be improved by optimizing the seed-layer deposition and the graphene transfer processes or by carefully annealing the devices.
